# Longitudinal associations between teacher–child relationships, resilience, and behavioral problems among preschool children

**DOI:** 10.3389/fpsyg.2026.1885244

**Published:** 2026-09-08

**Authors:** Lanlan Luo, Xiaoxia Fu, Shan Jin, Yanzhen Xu

**Affiliations:** 1 School of Educational Science, Guangdong Polytechnic Normal University, Guangzhou, China; 2 Business School, Central University of Finance and Economics, Beijing, China; 3 Institute for the Rule of Law and Standards in Education, China National Academy of Educational Sciences, Beijing, China

**Keywords:** behavioral problems, cross-lagged models, preschool children, resilience, teacher–child relationships

## Abstract

**Introduction:**

In preschool children, teacher–child relationships and psychological resilience—particularly its self-regulation component—have been identified as important correlates of behavioral problems. However, the nature of their relationship and the mechanism of their interaction still require further understanding and exploration.

**Methods:**

Drawing on teacher-reported questionnaires, this two-wave longitudinal study (*N* = 240 Chinese left-behind preschool children, 1-year interval) assessed teacher–child relationships (closeness and conflict), three facets of resilience (initiative, self-regulation, and attachment/relationships), and behavioral problems. Cross-lagged panel models were employed to examine the longitudinal associations between teacher–child relationships and resilience, and mediation analyses tested whether self-regulation mediated the link between teacher–child relationships and behavioral problems.

**Results:**

Contrary to expectations, the cross-lagged effects were unidirectional: children's resilience (as reported by teachers) was longitudinally associated with subsequent teacher–child relationships, but not the reverse. Self-regulation mediated both concurrent and longitudinal associations between teacher–child closeness/conflict and behavioral problems.

**Conclusion:**

These findings suggest that self-regulation may serve as a mechanism linking teacher–child relationship quality to behavioral outcomes in left-behind preschool children, although the correlational design precludes causal inferences.

## 1 Introduction

The current social and family environment is constantly changing, and the proportion of children and adolescents with behavioral problems is increasing, which has had noticeable adverse effects on learning quality and mental health. The Yuan (2024) conducted a survey on the mental health status of 47,563 children nationwide and found that children in China have a relatively high detection rate of depression risk and anxiety risk. There is an upward trend in emotional disorders and behavioral problems among children. Data shows that 14.9% of children are at risk of mild depression, and 4.9% are at risk of severe depression. Children with behavioral problems such as aggression, destructiveness, or lack of concentration often have difficulty following classroom rules, resulting in low learning efficiency and even disrupting the teaching order. If these problems are not intervened in time, they will not only aggravate the child's social difficulties but also lead to peer exclusion, further deteriorating their psychological state and learning performance (Ezmeci and Akman, 2023). Behavioral problems are primarily manifested as school and social phobia, destructive behavior, aggressive behavior, overactive behavior, and other symptoms that can adversely affect children's cognitive functions, interfere with their daily learning and social interactions, and cause a series of corresponding negative effects on their family and society (Aisyah et al., 2023). Childhood is a highly sensitive period for the coordinated development of an individual's psychology and physiology. Their emotional regulation system is not yet mature, and their cognitive assessment ability is significantly lower than that of adults. Therefore, external stress events from academic situations, family interactions, and peer relationships are more likely to be perceived as “threatening” stimuli, triggering intense negative emotional experiences. If there are no effective buffering resources, this emotional tension will be solidified into stable behavioral problems through externalization pathways (such as aggression, disciplinary violations) or internalization pathways (such as withdrawal, anxiety), thereby increasing the subsequent risk of psychological pathology. Furthermore, individuals who had behavioral problems during childhood have a significantly higher risk of developing psychological and behavioral disorders in adulthood. Specifically, they exhibit an increased tendency toward antisocial behavior (such as criminal activities), a higher incidence of substance dependence (such as drug abuse), and a higher prevalence of mental disorders (such as depression and anxiety disorders) compared to the general population. This phenomenon may be related to the social maladjustment that was linked to by early behavioral problems, abnormal neurodevelopment, and the cumulative effects of environmental risk factors. Therefore, early intervention for children's behavioral problems may become an important preventive strategy to reduce the incidence of psychological disorders in adulthood (Qiao et al., 2024). Therefore, paying more attention to and solving children's behavioral problems is crucial. Existing studies have shown that the main factors affecting behavior concerns include psychological (Tan et al., 2023), social (Aisyah et al., 2023; Zhu et al., 2024), and educational factors (Bulotsky-Shearer et al., 2020). In this context, the teacher–child relationship is vital among the educational factors affecting behavioral problems in children.

A particularly vulnerable subgroup is that of left-behind children—those whose parents have migrated to urban areas for work, leaving them in rural communities under the care of grandparents or other relatives for extended periods, typically 6 months or more. In China, rapid urbanization has resulted in a substantial population of such children, estimated at over 9 million in rural areas. These children face heightened psychosocial risks, including reduced parental supervision, limited emotional support, and economic disadvantage—factors linked to higher rates of behavioral and emotional problems compared to their non-left-behind peers (Yang et al., 2021; Tan et al., 2023). For these children, teachers often serve as primary stable adult attachment figures and may function as surrogate caregivers in ways that extend beyond the typical teacher role (Verschueren and Koomen, 2012). Consequently, the teacher–child relationship may be especially significant for these children's development, and understanding its associations with resilience and behavioral outcomes carries both theoretical and practical importance.

The teacher–child relationship refers to the psychological relationship generated by interactions between children and teachers, including two dimensions of closeness and conflict—its main manifestations include emotion, cognition, and behavior (Zhu et al., 2023). According to Pianta's (2001) conceptual framework, teacher–child closeness is characterized by warm and open communication, while conflict reflects tension, negativity, and a lack of rapport (Hu et al., 2023). This two-dimensional framework has become the dominant model in early childhood research (Spilt et al., 2022). This relationship is not only manifested in emotional opposition and resistance, but also shows detachment and resistance at the behavioral level. Long-term exposure to such negative interactions can make children feel neglected or invalidated, leading to negative emotions such as loneliness and inferiority. If these emotions are not promptly addressed, they may further affect their learning motivation and classroom participation, resulting in a decline in academic performance. At the same time, some children may express their inner unease and frustration through external behaviors, such as aggression and disobedience, thus falling into a vicious cycle and affecting their social adaptation and psychological health development (Lu et al., 2022). In addition, the daily interaction mode between teachers and students has a significant impact on the establishment of emotional connections between teachers and students. This kind of interaction is not limited to knowledge transmission in traditional teaching scenarios, but also involves multidimensional communication such as emotional support and behavioral guidance. When teachers can provide positive and stable interactions, students are more likely to form secure attachment relationships, and this emotional connection will further positively regulate students' classroom participation, learning motivation, and behavioral performance. The teacher–child relationship can affect the development of students' self-concept, emotional expression, school performance, and prosocial behavior. Therefore, it is crucial in solving children's behavioral problems.

In addition, as a positive psychological quality, psychological resilience directly affects children's mental health and can influence emotional and behavioral problems—good mental resilience can promote healthy growth and potential development. Moreover, a high psychological resilience can reduce the negative emotions produced by individuals during stressful events, thus improving their subjective wellbeing and quality of life. Existing studies have shown that psychological resilience is also associated with emotional feelings, problem behaviors, school participation, social skills, and the formation of a self-concept (Lonigan et al., 2017; Portilla et al., 2014; Yang et al., 2021). Psychological resilience, as the core ability of individuals to cope with adversity, mainly functions through the flexibility of resource mobilization and the cognitive restructuring with a growth-oriented perspective. Individuals with high psychological resilience can effectively integrate internal cognitive strategies (such as positive attribution) and external support systems (such as social networks), and activate adaptive coping patterns in stressful situations. This ability not only promotes the occurrence of posttraumatic growth (posttraumatic growth), by transforming challenges into development opportunities, but also significantly reduces the incidence of behavioral problems such as anxiety and aggression (Pietrzak et al., 2009; Troy and Mauss, 2011). For left-behind children in particular, psychological resilience may serve as a crucial protective factor. In the absence of consistent parental support, these children must rely more heavily on their own internal coping resources and on support from non-parental adults, such as teachers (Yang et al., 2021; Huang et al., 2019). Therefore, improving children's psychological resilience is of significant practical significance for addressing behavioral problems and improving teacher–child relationships.

In conclusion, exploring the interaction between teacher–child relationship quality, psychological resilience, and behavioral problems holds both theoretical and practical significance. This study focuses on left-behind preschool children to examine: (a) the bidirectional longitudinal associations between teacher–child relationships and resilience, and (b) whether resilience, particularly self-regulation, mediates the link between teacher–child relationships and behavioral problems. By investigating these associations in a population characterized by reduced parental support, the study can shed light on whether and how teacher–child relationships and resilience serve as protective factors for behavioral adjustment in high-risk contexts. Specifically, the study addresses the following research questions: (1) Are there bidirectional longitudinal associations between teacher–child relationships (closeness and conflict) and children's psychological resilience? (2) Does psychological resilience, especially self-regulation, mediate the association between teacher–child relationships and behavioral problems among left-behind preschool children?

## 2 Literature review and theoretical hypotheses

### 2.1 Teacher–child relationships and behavioral problems

According to Pianta's (2001) student–teacher relationship scale (STRS) framework, the quality of teacher–child relationships can be characterized along two primary dimensions: closeness and conflict. A substantial body of longitudinal research has demonstrated that these relational dimensions are associated with children's behavioral adjustment. Specifically, teacher–child closeness has been found to be negatively associated with both externalizing and internalizing behavioral problems (Berry and O'Connor, 2010; Collins et al., 2017; Paes et al., 2023), whereas teacher–child conflict has been positively linked to increased externalizing behaviors, low self-esteem, and poor academic achievement (Harvey et al., 2022; O'Connor et al., 2012; Skalická et al., 2015). O'Connor et al. (2012) found that increases in conflict were associated with heightened externalizing behaviors, while lower levels of closeness were related to internalizing behaviors. Skalická et al. (2015) further identified bidirectional associations between teacher–child conflict/closeness and children's behavioral problems.

Several mechanisms have been proposed to explain the protective role of teacher–child closeness. Close relationships provide children with a sense of emotional security, enabling them to explore their social environment with confidence (Verschueren, 2015). In such relationships, children observe teachers' behavior, imitate adaptive communication patterns, and gradually learn to express their opinions and feelings appropriately. Research suggests that the quality of emotional connections between teachers and children serves as a core proximal factor associated with multiple developmental indicators in preschool and school-aged children, including learning motivation, social-emotional skills, and positive peer relationships (Lu et al., 2022; Hu et al., 2023). Moreover, when children develop a trusting relationship with their teachers, they are more willing to openly express their feelings and needs, facilitating early detection and intervention for emerging behavioral difficulties (Spilt et al., 2022). Conversely, teacher–child conflict is linked to negative developmental outcomes. When interactions are characterized by adversarial, indifferent, or highly controlling patterns, children's basic psychological needs (e.g., autonomy, belongingness, and competence) are less likely to be satisfied, which may activate stress responses and impair emotional regulation and behavioral control (Harvey et al., 2022). Such conflictual relationships may lead children to perceive adults as unreliable, fostering a negative bias toward teaching behaviors and increasing behavioral problems. Recurring conflicts with teachers have been associated with adverse effects on children's school performance over time (Spilt et al., 2022). Collins et al. (2017) found that teacher–child relationship quality was significantly associated with behavior problems among low-income boys in elementary school, underscoring the role of conflict as a risk factor.

In summary, both cross-sectional and longitudinal studies indicate that teacher–child closeness serves as a protective factor, while teacher–child conflict acts as a risk factor for children's behavioral adjustment (Berry and O'Connor, 2010; Collins et al., 2017; Harvey et al., 2022; O'Connor et al., 2012; Paes et al., 2023; Pianta, 2001; Skalická et al., 2015). Building on this evidence, the following hypotheses are proposed:

Hypothesis 1. Teacher–child closeness negatively associated with behavioral problems.
Hypothesis 2. Teacher–child conflict positively associated with behavioral problems.

### 2.2 Teacher–child relationships and psychological resilience

Psychological resilience refers to individuals' rapid adaptation process and psychological rebound ability during stressful events, including coping strategies and a positive mentality, mainly reflected in initiative, self-regulation, and attachment/relationship (Veríssimo et al., 2017). Closeness teacher–child relationships provide more opportunities for children to interact with teachers, and frequent, personalized, and well-coordinated verbal interactions may occur more often (Sergienko and Ulanova, 2016). Children gradually build up self-confidence through positive interaction with teachers, which can catalyze the formation of a positive self-concept and improve children's psychological resilience. Moreover, harmonious, free, and warm relationships can encourage children to actively communicate with teachers, enhance their autonomy, improve their self-regulation ability, and optimize their evaluation of themselves. At the same time, high-quality teacher–student relationships are regarded as a key proximal resource for the development of children's psychological resilience. When teachers build close interactions through high emotional responsiveness, high autonomy support, and continuous positive feedback, children experience a sense of security and belonging in the classroom, thereby enhancing their psychological resilience in the face of stressful events (Zhang et al., 2023).

In contrast, in teacher–child conflict, children may be criticized, blamed, and ignored by teachers, increasing feelings of helplessness and depression in the face of difficulties. This situation may cause children to doubt their ability and value, affecting their self-perception (Rudasill et al., 2022). Such relationships can also cause negative emotions, such as depression and anger. So, children may become reluctant to communicate with teachers or other children, potentially leading to social avoidance behavior. The long-term accumulation of negative cognition and emotions linked to teacher–child conflict can weaken children's psychological resilience, making it difficult for them to cope with future challenges and difficulties (Rudasill et al., 2022; Spilt et al., 2022).

Furthermore, psychological resilience is a key factor affecting children's mental health development, and it is an important ability for individuals to cope with adversity. Children with high psychological resilience can find internal and external resources during adversity and make efficient use of these resources. They have strong psychological adaptability, can easily understand and accept, and can better regulate their emotions, improving the closeness between teachers and students and reducing conflicts (Portilla et al., 2014). For example, when individuals have high psychological resilience, they also have substantial initiative and self-regulation ability. When facing adverse, stressful events, the influence of negative cognition can be reduced, significantly improving the individual's social ability, reducing anxiety, and improving the quality of teacher–child relationships (Dias and Cadime, 2017). For left-behind children, who often receive limited emotional support from their parents, teachers may serve as especially crucial sources of relational security, thereby fostering resilience development (Yang et al., 2021).

Moreover, psychological resilience can affect and regulate the impact of adverse life events. The better the psychological resilience, the lower the sensitivity to stressful events, and the better the ability to understand problems from different perspectives, avoid overreaction or emotional loss, and try various solutions to deal with teacher–child conflicts more peacefully and rationally. The faster children recover from negative life events, the better their school adaptability and relationship with teachers, and the more they can improve their learning engagement (Huang et al., 2019). In contrast, children with weak psychological resilience are prone to anxiety, fear, and anger; such children may struggle to control their emotions and are prone to more aggressive or defensive behaviors when facing external pressure. This situation increases the tension between teachers and children and may further escalate the conflict. Moreover, children with weak psychological adaptation skills lag significantly in the dimensions of cognitive reappraisal and emotional expression. When facing classroom rules or assignment requirements, they often adopt vague, withdrawn, or even confrontational expressions due to their inability to accurately identify their own needs. This inefficient communication mode easily leads teachers to make attribution biases, such as uncooperative or deliberate violation of rules, and subsequently, teachers adopt more severe control strategies. As the frequency of negative interactions increases, the emotional synchronization between teachers and students gradually breaks down, ultimately amplifying the conflict intensity and solidifying problem behaviors.

In summary, close teacher–child relationships may foster children's psychological resilience by offering emotional security and positive interaction experiences, whereas conflictual relationships can undermine resilience through chronic negative affect and diminished self-worth (Rudasill et al., 2022; Zhang et al., 2023). Conversely, children with higher psychological resilience tend to demonstrate better emotional regulation and social adaptability, which may facilitate more positive interactions with teachers and reduce interpersonal conflicts (Pietrzak et al., 2009; Huang et al., 2019; Portilla et al., 2014). For left-behind children, psychological resilience may be especially crucial in maintaining positive teacher–child relationships. Research on Chinese left-behind children has revealed significant associations between resilience and social adaptability (Pietrzak et al., 2009; Huang et al., 2019), and those with higher resilience are more adept at forming positive interpersonal relationships and adapting to changes in the school environment. Although theoretical arguments support bidirectional influences between teacher–child relationships and resilience, longitudinal empirical evidence for the direction from teacher–child relationships to subsequent resilience remains scarce. Therefore, the present study empirically tests this bidirectional hypothesis rather than assuming it to be true. Accordingly, this study proposes the following hypothesis:

Hypothesis 3. Teacher–child relationships and psychological resilience are reciprocally associated over time.

### 2.3 Mediating role of psychological resilience

A substantial body of evidence supports the association between the quality of teacher–child relationships and children's behavioral problems (Berry and O'Connor, 2010; Collins et al., 2017; Harvey et al., 2022; O'Connor et al., 2012; Paes et al., 2023). Building on this foundation, the present study investigates whether psychological resilience—specifically self-regulation—mediates this relationship.

Interpersonal relationships are one of the key factors affecting psychological resilience. The support, care, and sense of security teachers provide can enhance children's inner strength and promote psychological resilience to some extent. The teacher–student relationship, as the core link in the process of children's socialization, its quality directly affects the cognitive construction and emotional development of students. The harmonious interaction between teachers and students serves as a buffer layer for psychological development. It not only helps children relieve academic pressure but also provides them with a model for social interaction. The value of this kind of relationship resource is not only reflected in the immediate emotional support, but also gradually internalized into the children's psychological capital for coping with challenges through the positive interactions accumulated over time (Lu et al., 2022). Children who establish a close relationship with teachers are more likely to obtain support, encouragement, and affirmation, and there may be more frequent, personalized, and well-coordinated verbal interactions between teachers and children, thus enhancing self-confidence and coping ability while reducing behavioral problems (Rudasill et al., 2022; Ceylan and Asi, 2022).

Furthermore, psychological resilience is also a series of protective measures and behaviors taken by individuals against stress to make themselves less negatively affected. Individuals with high psychological resilience can cope with external pressure to avoid psychological imbalance (Dias and Cadime, 2017). High-quality teacher–child relationships are important sources of psychological resilience that support students' exploration of the environment, improving their psychological resilience, increasing positive emotions, promoting cognitive development, and helping them cope with daily challenges, thus cultivating their adaptability and high psychological resilience (Rudasill et al., 2022). Individuals with high psychological resilience have low levels of anxiety and high levels of self-acceptance when facing stressful events. In most cases, they can manage thoughts and maintain appropriate behavior, relieve their social anxiety, cooperate with peers, and strive to achieve their goals; control their cognition, motivation, and behavior; and thus reduce the frequency of their behavioral problems (Ezmeci and Akman, 2023).

In contrast, teacher–child conflict can negatively affect psychological resilience, leading children to develop or intensify maladaptive behaviors and reactions. The lower their psychological resilience, the greater the likelihood of self-harm, conflict, and resistance. For instance, when the closeness (or conflict) of a key factor influencing children's growth and development is weak (or high), children's initiative, self-regulation, and attachment or relational quality tend to be relatively low (Housman et al., 2023). This situation results in children's inability to detect, identify, and regulate their own emotions as well as those of others and the environment, leading to more behavioral problems (Rudasill et al., 2022). In the negative environment of teacher–child relationships, children often have limited development of receptive language ability, and weak receptive language ability may be a chronic and continuous pressure. This situation can impair children's psychological resilience and affect their cognition and self-evaluation, resulting in behavioral problems. Based on the evidence reviewed above, psychological resilience may serve as a mediating factor in the link between teacher–child relationships and behavioral problems. Close teacher–child relationships have been positively associated with children's resilience—encompassing initiative, self-regulation, and relational capacity (Rudasill et al., 2022; Zhang et al., 2023)—which in turn has been negatively associated with behavioral problems (Lonigan et al., 2017; Dias and Cadime, 2017; Ezmeci and Akman, 2023). Conversely, teacher–child conflict has been negatively associated with resilience, potentially contributing to increased behavioral difficulties (Housman et al., 2023; Rudasill et al., 2022). It should be noted that this reasoning is largely based on concurrent associations; whether such mediation operates prospectively remains to be conclusively established. The present study empirically tests this possibility by examining all three facets of resilience as potential mediators. Therefore, this study proposes the following hypothesis:

H4. Psychological resilience mediates the relationship between teacher–child closeness and behavioral problems.
H5. Psychological resilience mediates the relationship between teacher–child conflict and behavioral problems.

## 3 Method

### 3.1 Participants

This study's sample included 240 children (M = 58.56 months, 55.8% boys) recruited from 16 classrooms in 8 preschools in China, who were left-behind children for at least 6 months. Data were collected over two waves, and the interval was 1 year. The average teacher–child ratio of these classes was 1:16. The sample was predominantly from low-income families, and the average monthly family income was 120.94 USD (Median: $ −36.03 to $5,887.5). The total annual household income varied significantly, with the minimum income falling below the poverty threshold. Their parents were aged 20–45 (SD = 4.694); 82.9% were engaged in non-technical work, and 17.1% were engaged in technical work. Parental educational attainment was distributed as follows: no vocational education (23.5%), junior middle school (60.3%), and senior middle school (16.2%). In this study, all 240 participating children met the criterion of having been left behind for at least 6 months at the time of initial enrollment. At both data collection points (T1 and T2), the children remained in their left-behind status, with their parents continuing to work in other cities. During this period, the primary caregivers were predominantly grandparents (78.3%), while the remaining children were cared for by other relatives (21.7%).

A total of 32 teachers participated in this study (two teachers per classroom). The participating teachers from the 16 classrooms were all female (100%), between 23 and 42 years old (SD = 6.76), and most of the teachers were experienced, with 62% having taught kindergarten for more than 4.86 years. The education background was mainly secondary school and below (71.15%). The teachers had no significant differences regarding average age, race/ethnicity, or gender.

### 3.2 Procedure

Data were collected from participants at two points: T1, preschool fall, and T2, preschool fall the following year. Active informed consent was obtained from all participating parents and teachers on-site prior to data collection. Survey packages containing consent forms and questionnaires were distributed to parents and teachers on-site by the research team during scheduled school visits. Parents who consented to participate completed a demographic survey regarding their children's sex, their profession, and the family's annual household income. Data were collected in the fall of the first year. The research team was present on-site to address any questions from parents and teachers regarding the survey completion. In addition, teachers were requested to complete their digital questionnaires assessing teacher–child relationships and structured questionnaires on children's resilience and behavioral problems during the visit. The same data were collected in the second year.

The research team consisted of five members, including the authors and two trained research assistants. Teachers completed a separate questionnaire for each child in their classroom, providing child-specific ratings of teacher–child relationship quality, resilience, and behavioral problems. A 1-year interval was chosen based on prior longitudinal research on teacher–child relationships in early childhood (e.g., Berry and O'Connor, 2010; Skalická et al., 2015), which indicates that relational and behavioral changes typically unfold over the course of an academic year. This 12-month lag allows sufficient time for cross-lagged effects to emerge while remaining within a single developmental period, and aligns with the time intervals used in comparable longitudinal studies of preschool children's social-emotional development (Portilla et al., 2014; Yang et al., 2021).

### 3.3 Measures

#### 3.3.1 Facets of the teacher–child relationship

Two dimensions of teacher–child relationships (closeness and conflict) were measured via teacher-reported questionnaires using the Student–Teacher Relationship Scale-Short Form (STRS) in two waves (Pianta, 2001). STRS is a widely used measure for children in preschool and elementary years. The closeness subscale in this study included seven items to assess the teachers' perceptions of students' warmth and willingness to communicate (e.g., the child cherishes his [her] relationship with me). Meanwhile, the conflict subscale consisted of eight items reflecting teachers' perceptions of conflicting relationships with the students (e.g., the child easily gets mad at me). Teachers rated children on the items using a 5-point Likert scale (1 = definitely does not apply; 3 = neutral/not sure; 5 = definitely applies). The items were summed for both subscales to create mean scores; higher scores indicate higher closeness or conflict. The internal consistency at T1 and T2 was adequate (T1, α = 0.719, and T2, α = 0.831 for closeness; T1, α = 0.794, and T2, α = 0.845 for conflict). The Chinese version of the STRS was adapted following a standard translation-back-translation procedure. The translation was conducted by two bilingual developmental psychologists, and the back-translation was reviewed by an independent English-speaking researcher to ensure semantic equivalence. The Chinese STRS has been validated in Chinese preschool samples with acceptable reliability and factorial structure (Hu et al., 2023; Zhu et al., 2023).

#### 3.3.2 Facets of resilience

Children's resilience was assessed with the Devereux Early Childhood Assessment for Preschoolers Second Edition (DECA-P2) (LeBuffe and Naglieri, 2012). The DECA-P2 is a behavior rating scale completed by teachers that assesses within-child protective factors that are central to social and emotional health and resilience. All the domains had high reliability with coefficients of 0.80 or above. The Chinese adaptation of these subtests has also demonstrated high reliability and validity. The measure is rated on a 5-point Likert scale (1 = never; 3 = occasionally; 5 = very frequently), with higher scores indicating greater resilience. Each subscale of the DECA-P2 is described below. The Chinese adaptation of the DECA-P2 was developed using standard translation-back-translation procedures. Two bilingual researchers independently translated the items into Chinese, and discrepancies were resolved through discussion. A back-translation was then conducted by a third bilingual researcher who was unfamiliar with the original items. The Chinese version has shown satisfactory reliability (Cronbach's α > 0.73 for all subscales) and construct validity among Chinese preschool children (Yang et al., 2021). It is important to note that the attachment/relationships dimension of the DECA-P2 measures children's general relational capacity across multiple contexts (e.g., peers, family members, and teachers), which is conceptually broader than the teacher–child relationship quality assessed by the STRS. Although both constructs involve relational components, the STRS specifically captures the dyadic quality of one particular relationship, whereas the DECA-P2 assesses the child's overall relational competence as a dispositional protective factor (LeBuffe and Naglieri, 2012; Pianta, 2001).

**Initiative** is one of the main protective factors for the resilience of left-behind children, referring to their ability to use independent thought and action to meet their needs (LeBuffe and Naglieri, 2012). Nine items were used to measure this factor. Sample questions included “try or ask to try new things or activities” and “remember important information.” This measure demonstrated good internal consistency at each time point. The Cronbach's α coefficients were 0.802 and 0.799 in waves 1 and 2, respectively.

**Self-regulation** is one of the main protective factors for the resilience of left-behind children, referring to their ability to express emotions and manage behaviors in a healthy way (LeBuffe and Naglieri, 2012). Nine items were used to measure this factor. Sample questions included “handle frustration well” and “listen to or respect others.” This measure demonstrated good internal consistency at each time point. The Cronbach's α coefficients were 0.842 and 0.796 in waves 1 and 2, respectively.

**Attachment/relationship** is one of the main protective factors for the resilience of left-behind children, referring to their ability to promote and maintain mutual, positive connections with other children and significant adults (LeBuffe and Naglieri, 2012). Nine items were used to measure this factor. Sample questions included “show affection for familiar adults” and “ask adults to play with or read to him/her.” This measure demonstrated good internal consistency at each time point. The Cronbach's α coefficients were 0.734 and 0.759 at waves 1 and 2, respectively.

#### 3.3.3 Facets of behavioral problems

Teachers reported about behavioral problems using the behavioral problems subscale of the DECA-P2 (LeBuffe and Naglieri, 2012), because parents of left-behind children are not around for a long time, and preschool is the second-most important place in children's daily lives. Teachers and left-behind children have more opportunities to get along. DECA-P2 is a 16-item measure rated on a 5-point Likert scale (1 = never; 3 = occasionally; 5 = very frequently; T1, α = 0.828; T2, α = 0.77). Sample questions include “damage to the property” and “have a temper tantrum.” The scale is widely used in China and has demonstrated good psychometric properties.

Participant retention and missing data: A total of 252 children were initially enrolled at T1. At T2, 240 children (95.2%) provided valid data and were retained for analysis. Attrition was primarily due to family relocation (*n* = 8) and absence on the day of data collection (*n* = 4). Comparison of completers and non-completers revealed no significant differences in T1 demographic variables (gender, age) or key study variables (teacher–child closeness, conflict, resilience, behavioral problems; all *p* > 0.10), suggesting that attrition was largely random. Missing data were handled using full information maximum likelihood (FIML) estimation in Mplus, which utilizes all available data and produces unbiased estimates under the assumption that data are missing at random (MAR).

### 3.4 Analyses

Given that only two waves of data were available, the conventional Cross-Lagged Panel Model (CLPM) was adopted for analysis (Mackinnon et al., 2022). We used a structural equation modeling (SEM) approach in Mplus version 8.3 to examine reciprocal associations between teacher–child relationships and children's resilience. To test the mediating role of resilience in the association between teacher–child relationships and behavioral problems, we conducted mediation analysis in Mplus 8.3. This analysis allowed for the inclusion of multiple mediating and control variables and provided estimates of the total, direct, and indirect effects. A total of 2,000 samples were selected in the bootstrap analysis with a 95% confidence interval, and the roles of resilience in teacher–child relationships and behavioral problems were analyzed.

## 4 Results

Descriptive statistics and bivariate correlations for the key variables at each wave are summarized in Tables 1, 2. Model fit indices for the tested models are displayed in Table 3, while the results of the mediation analysis are presented in Table 4.

**Table 1:** Descriptive statistics for child resilience and teacher–child relationships.

Variables	N	M	SD	Range
Teacher–child closeness				
1. Wave 1	216	23.77	0.251	12–32
2. Wave 2	225	23.64	0.285	9–33
Teacher–child conflict				
3. Wave 1	226	14.87	0.299	9–30
4. Wave 2	226	17.26	0.342	9–33
Initiative				
5. Wave 1	235	25.80	0.349	12–40
6. Wave 2	240	27.71	0.358	12–43
Self-regulation				
7. Wave 1	237	29.93	27.81	14–45
8. Wave 2	240	32.05	0.337	15–45
Attachment/relationships				
9. Wave 1	239	31.37	0.306	16–44
10. Wave 2	240	32.05	0.340	19–45
Behavioral problems				
11. Wave 1	240	34.8833	0.39188	16–47
12. Wave 2	240	25.03	0.357	11–44

**Table 2:** Bivariate correlations between child resilience, teacher–child relationships, and behavioral problems.

Variables	1	2	3	4	5	6	7	8	9	10	11
Teacher–child closeness											
1. Wave 1											
2. Wave 2	0.232^**^										
Teacher–child conflict											
3. Wave 1	−0.268^**^	−0.130									
4. Wave 2	−0.057	−0.245^**^	0.210^**^								
Initiative											
5. Wave 1	0.419^**^	0.199^**^	−0.187^**^	−0.166^*^							
6. Wave 2	0.097	0.427^**^	−0.061	−0.015	0.086						
Self-regulation											
7. Wave 1	0.397^**^	0.182^**^	−0.364^**^	−0.266^**^	0.670^**^	0.103					
8. Wave 2	0.005	0.275^**^	−0.108	−0.252^**^	0.076	0.649^**^	0.174^**^				
Attachment/relationships											
9. Wave 1	0.415^**^	0.306^**^	−0.129	−0.172^**^	0.597^**^	0.031	0.467^**^	0.029			
10. Wave 2	0.038	0.406^**^	−0.072	−0.039	0.020	0.563^**^	0.029	0.502^**^	0.091		
Behavioral problems											
11. Wave 1	−0.0513^**^	0.044	−0.162^*^	0.056	−0.193^**^	−0.026	−0.146^*^	0.000	−0.148	−0.148^*^	−0.096
12. Wave 2	−0.011	0.218^**^	−0.002	0.309^**^	−0.095	−0.256^**^	−0.216^**^	−0.399^**^	0.010	−0.272^**^	−0.337^**^

^**^Significant correlation at level of 0.01 (bilateral), ^*^Significant correlation at level of 0.05 (bilateral).

**Table 3:** Cross-lagged path models assessing the directional associations between child resilience and teacher–child relationships.

Model	χ^2^ (df)	CFI	TLI	RMSEA	SRMR	Scaling correction factor
Initiative						
1. M1	48.281 (20)	0.852	0.741	0.049	0.079	1.0232
2. M2	41.258 (18)	0.878	0.763	0.044	0.074	1.0274
Self-regulation						
1. M1	66.743 (20)	0.773	0.603	0.073	0.087	1.0444
2. M2	55.023 (16)	0.811	0.586	0.072	0.079	1.0579
Attachment/relationships						
1. M1	50.625 (20)	0.836	0.712	0.053	0.075	1.0088
2. M2	30.250 (16)	0.924	0.833	0.025	0.064	1.0017

M1, stability; M2, reciprocal.

**Table 4:** Results of the mediation effect analysis.

Path	β	*p*	Effects' 100%	Bootstrap 95% CI	
				Lower	Upper
1. T1 teacher–child closeness–T1 behavioral problems	−1.423	0.000	–	−1.869	−0.978
2. T1 teacher–child closeness–T1 self-regulation–T2 behavioral problems	−0.108	0.035	100	−0.192	−0.024
3. T1 teacher–child conflict–T2 behavioral problems	0.189	0.044	–	0.035	0.344
4. T1 teacher–child conflict–T1 self-regulation–T2 behavioral problems	0.083	0.042	30.51	0.016	0.150
5. T2 teacher–child conflict–T2 behavioral problems	0.244	0.000	–	0.131	0.358
6. T2 teacher–child conflict–T2 self-regulation–T2 behavioral problems	0.063	0.018	20.45	0.019	0.104

### 4.1 Cross-lagged analysis of teacher–child relationships and children's resilience

#### 4.1.1 Initiatives and teacher–child relationships

**Figure 1:**
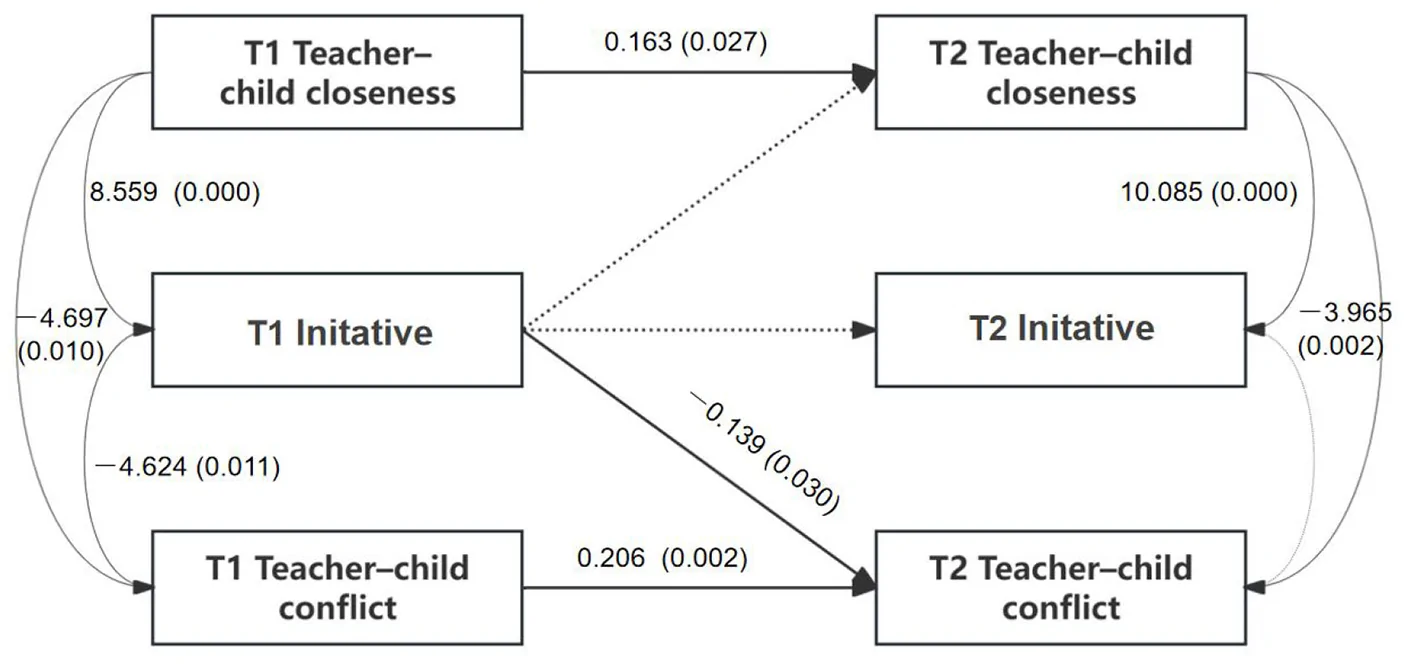
Relationships among initiative, closeness, and conflict at wave 1–2. All parameters are standardized coefficients, and significant coefficients. IN, initiative.

Table 3 shows that the two models testing the within-time and prospective associations between child initiative and teacher–child relationships fit the data well. M2 demonstrated a fit that was acceptable to the data. The model fit of M2 was acceptable: χ^2^/df = 2.29 (<3), CFI = 0.878, TLI = 0.763, RMSEA = 0.077, and SRMR = 0.074. Consistent with the bivariate correlations, weak stability exists in the teacher–child relationships autoregressive paths (Figure 1). Children's initiative in wave 1 significantly and negatively predicted subsequent teacher–child conflict in wave 2 (β = −0.139, SE = 0.064, and *p* < 0.05); however, the initiative in wave 1 did not predict teacher–child closeness in wave 2. All the autoregressive paths in M2 were statistically significant. We acknowledge that the CFI (0.878) and TLI (0.763) fall below the conventional threshold of 0.95 (Hu and Bentler, 1999). However, Marsh et al. (2004) cautioned that these cutoff values were derived under specific conditions and should not be treated as universal “golden rules.” CFI and TLI tend to be systematically deflated when the sample size is relatively small and the model complexity is high (Shi et al., 2018). Given N = 240 and the complexity of the cross-lagged specification, some deflation is expected. The RMSEA (0.077) and SRMR (0.074) are within acceptable ranges (≤ 0.08), suggesting reasonable model-data correspondence. Following the recommendation to evaluate fit holistically (Marsh et al., 2004), we conclude that the model fit is acceptable.

#### 4.1.2 Self-regulation and teacher–child relationships

**Figure 2:**
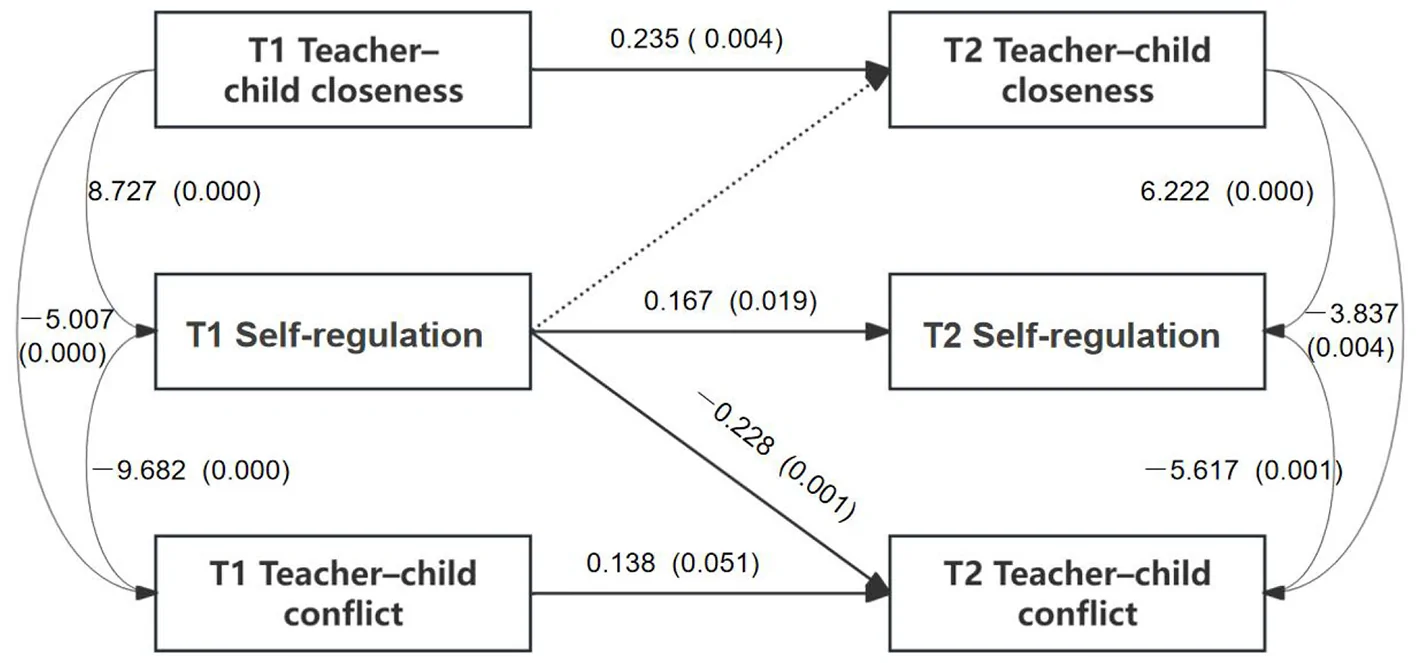
Relationships among self-regulation, closeness, and conflict at wave 1–2. All parameters are standardized coefficients, and significant coefficients. S-R, self-regulation.

Table 3 shows that the two models testing the within-time and prospective associations between child self-regulation and teacher–child relationships fit the data well. M2 demonstrated a fit that was acceptable to the data. The model fit of M2 was acceptable: χ^2^/df = 3.44 (<5), CFI = 0.811, TLI = 0.586, RMSEA = 0.072, and SRMR = 0.079. Consistent with the bivariate correlations, weak stability exists in the teacher–child relationships and self-regulation autoregressive paths (Figure 2). Children's self-regulation in Wave 1 significantly and negatively predicted subsequent teacher–child conflict in Wave 2 (β = −0.228, SE = 0.069, and *p* < 0.001); however, self-regulation in Wave 1 did not predict teacher–child closeness in Wave 2. All the autoregressive paths in M2 were statistically significant. The CFI (0.811) and TLI (0.586) are below conventional thresholds. However, Marsh et al. (2004) demonstrated that rigid application of cutoff values can lead to rejection of substantively meaningful models with modest sample sizes. Simulation studies show that incremental fit indices are negatively biased in small-N complex models (Shi et al., 2018). The absolute fit indices—RMSEA (0.072) and SRMR (0.079)—are within acceptable ranges, indicating the discrepancy between model-implied and observed covariance matrices is not severe. We interpret the fit as marginally acceptable based on holistic evaluation.

#### 4.1.3 Attachment/relationships and teacher–child relationships

**Figure 3:**
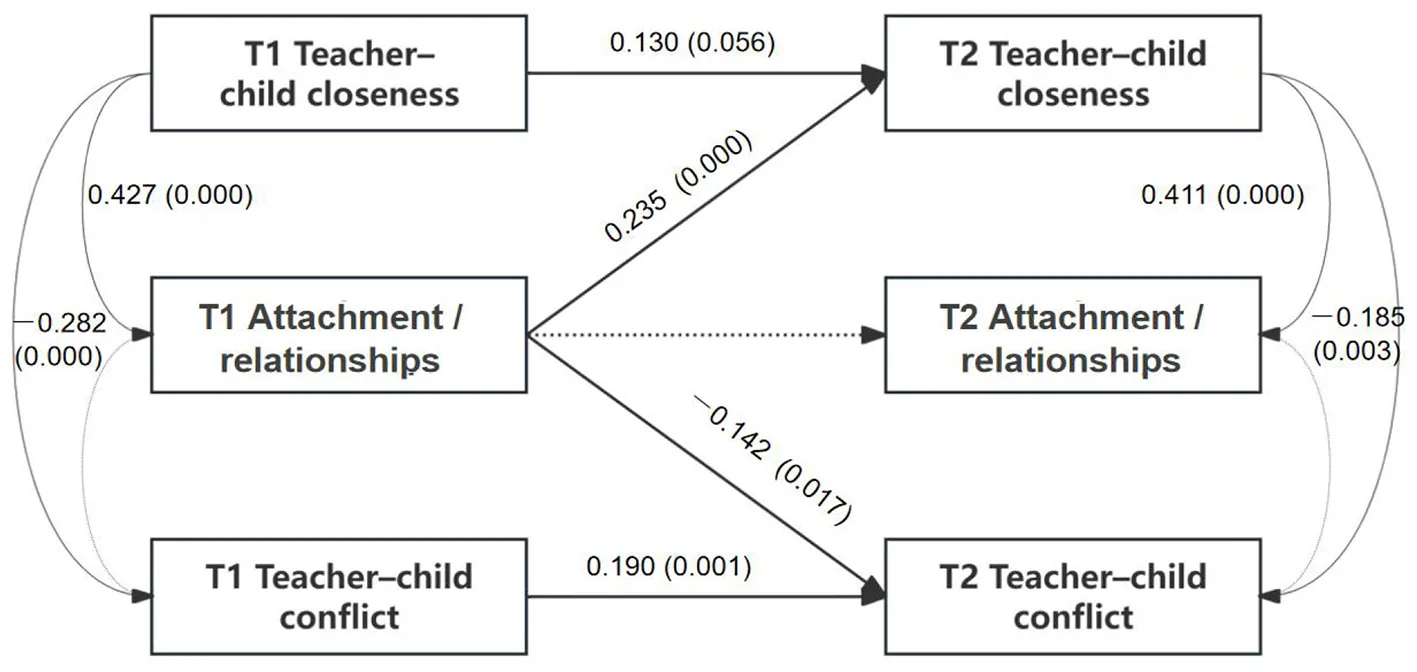
Relationships among attachment / relationships, closeness, and conflict at wave 1–2. All parameters are standardized coefficients and significant coefficients. A/R, attachment/relationships.

Table 3 shows that the two models testing the within-time and prospective associations between child attachment/relationships and teacher–child relationships fit the data well. M2 provided the best fit to the data. The model fit of M2 was acceptable (χ^2^/df = 1.89 (<3), CFI = 0.924, TLI = 0.833, RMSEA = 0.061, and SRMR = 0.064). Consistent with the bivariate correlations, weak stability exists in the teacher–child relationships and attachment/relationships autoregressive paths (Figure 3). Children's attachment/relationships in Wave 1 significantly and positively predicted subsequent teacher–child closeness in Wave 2 (β = 0.213, SE = 0.055, and *p* < 0.001). Moreover, the attachment/relationships in wave 1 significantly and negatively predicted teacher–child conflict in wave 2 (β = −0.153, SE = 0.062, and *p* < 0.05). All the autoregressive paths in M2 were statistically significant.

### 4.2 Mediation analysis for teacher–child relationships, resilience, and children's behavioral problems

Using the teacher–child relationship as the independent variable, child behavioral problems as the dependent variable, and resilience (initiative, self-regulation, and attachment/relationships) as the mediating variable, it was determined that self-regulation exhibited a significant correlation with both the teacher–child relationship and the child's behavioral problems (Alfons et al., 2021).

#### 4.2.1 Self-regulation, teacher–child closeness, and behavioral problems

**Figure 4:**
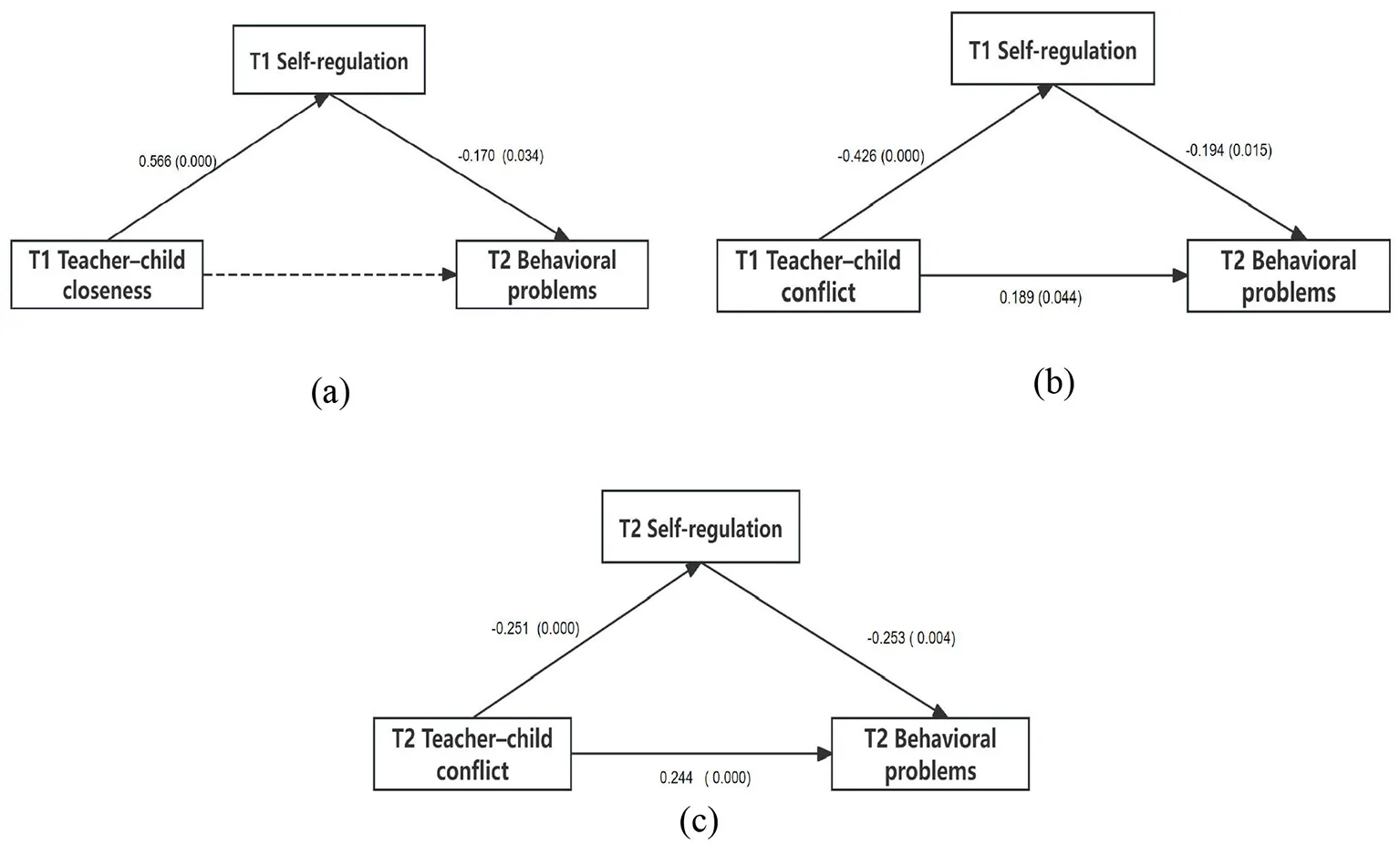
Relationships among behavioral problems, teacher–child relationship, and self-regulation at wave 2. Path coefficients are standardized; dashed lines indicate non-significant path coefficients. Gender and age were controlled for.

Figure 4a shows that after controlling for gender and age, teacher–child closeness in wave 1 was significantly and positively related to self-regulation in wave 1 (β = 0.566, SE = 0.098, and *p* < 0.001). Self-regulation in Wave 1 was significantly and negatively related to behavioral problems in Wave 1 (β = −0.191, SE = 0.080, and *p* < 0.005). The bootstrap test showed a significant indirect effect of teacher–child closeness in wave 1 on behavioral problems in wave 2 through self-regulation in wave 1 (β = −0.108, SE = 0.051, and *p* < 0.005) with a 95% confidence interval of [−0.192, −0.024] (Table 4). The results indicate that teacher–child closeness in wave 1 is related to behavioral problems in wave 2 through their association with self-regulation in wave 1.

#### 4.2.2 Self-regulation, teacher–child conflict, and behavioral problems

Figure 4b shows that after controlling for gender and age, teacher–child conflict in wave 1 was significantly and negatively related to self-regulation in wave 1 (β = −0.426, SE = 0.092, and *p* < 0.001), which, in turn, was significantly and positively related to behavioral problems in wave 1 (β = 0.189, SE = 0.094, and *p* < 0.05). Self-regulation in Wave 1 was significantly and negatively related to behavioral problems in Wave 2 (β = −0.194, SE = 0.080, and *p* < 0.05). The bootstrap test showed a significant indirect effect of teacher–child conflict in wave 1 on behavioral problems in wave 2 through self-regulation in wave 1 (β = 0.083, SE = 0.041, and *p* < 0.05) with a 95% confidence interval of (0.016, 0.150). These results indicate that teacher–child conflict in wave 1 is partly related to behavioral problems in wave 2 through their association with self-regulation in wave 1.

Figure 4c shows that after controlling for gender and age, teacher–child conflict in wave 2 was significantly and negatively related to self-regulation in wave 1 (β = −0.251, SE = 0.060, and *p* < 0.001), which, in turn, was significantly and positively related to behavioral problems in wave 2 (β = 0.244, SE = 0.069, and *p* < 0.001). Self-regulation in Wave 2 was significantly and negatively related to behavioral problems in Wave 2 (β = −0.253, SE = 0.088, and *p* < 0.05). The bootstrap test showed a significant indirect effect of teacher–child conflict in wave 2 on behavioral problems in wave 2 through self-regulation in wave 2 (β = 0.063, SE = 0.027, and *p* < 0.05) with a 95% confidence interval of (0.019, 0.108). These results indicate that teacher–child conflict in wave 2 is partly related to behavioral problems in wave 2 through their association with self-regulation in wave 2.

## 5 Discussion

Based on the two-wave panel data, we examined the bidirectional relationships between teacher–child relationships (closeness and conflict) and resilience (initiative, self-regulation, and attachment/relationships) in estimating the cross-lagged effects. Furthermore, we examined resilience as a mediator between teacher–child relationships and behavioral problems. We controlled for age and gender in the analyses. Our findings did not support the bidirectional relationship between teacher–child relationships and resilience. Instead, we found significant cross-lagged effects from resilience to teacher–child relationships only. Children who performed better in resilience displayed lower levels of teacher–child conflict and higher levels of teacher–child closeness over time. The mediating effect of resilience (initiative, self-regulation, and attachment/relationships) is significant only for self-regulation. The cross-lagged results did not support a prospective effect of teacher–child relationships on resilience, which may appear inconsistent with our mediation findings. However, the former assessed change over 1 year, while the latter captured associations within the same wave. The significant mediation paths indicate that, at a given time point, closer teacher–child relationships co-occur with better self-regulation, which in turn co-occurs with fewer behavioral problems. Therefore, the mediation should be interpreted as concurrent associative rather than causal.

We explicitly acknowledge that Hypothesis 3 received only partial support. The predicted bidirectional association was not confirmed; instead, only the path from resilience to subsequent teacher–child relationships emerged as significant. This finding suggests that, contrary to our theoretical expectations grounded in attachment theory, teacher–child relationship quality may not prospectively foster resilience development within the studied timeframe. Rather, children who already possess stronger resilience resources appear to elicit more positive interactions from teachers. This child-driven effect aligns with evocative person–environment correlation models (Scarr and McCartney, 1983), in which children's characteristics actively shape their social environments. Future research should explore whether teacher–child relationships might influence resilience over longer developmental periods or through mechanisms distinct from those examined here.

### 5.1 Relationships between teacher–child relationships and children's resilience

Children's resilience in teacher–child relationships has received relatively little attention compared to the role of teacher–child relationships. Our findings seem to highlight the contribution of resilience to teacher–child relationships rather than the opposite direction. Although empirical evidence is still limited, these findings underscore the role of resilience in teacher–child relationships. The theory of mind partly shapes a close teacher–child relationship, and it represents a mechanism of social cognition that provides communicative success between others through understanding each other's mentality (Sergienko and Ulanova, 2016). Therefore, children with greater resilience are more inclined to develop positive interpersonal relationships in interpersonal situations.

Specifically, children's attachment/relationships have a positive predictive effect on teacher–child closeness and a negative predictive effect on teacher–child conflict; this finding is consistent with related empirical evidence. According to attachment theory, teachers are “adult attachment figures” (Zajac and Kobak, 2006), particularly in kindergarten settings. They provide both a safe haven and a secure base for children, as reflected in initially high scores for security-seeking behavior at school entry, followed by a marked decrease in such behaviors over the subsequent weeks (Verschueren and Koomen, 2012). Most studies have confirmed the role of teachers' attachment to children (Verschueren, 2015), but not the opposite relationship. Secure attachment representations play a key role in fostering the development of a positive and close teacher–child relationship, which may be responsible for the predictive effect of child attachment on teacher–child relationships. That is, it could be that more secure attachment representations enable children to initiate contact with their teachers to inquire about classroom activities and play opportunities or to adjudicate disputes more frequently than children with less secure attachment representations (Veríssimo et al., 2017). Our findings confirm that establishing positive relationships with teachers is equally influenced by the children's representations of attachment and positive relationships.

Our results suggest that self-regulation was negatively longitudinally associated with teacher–child conflict, but the relationship with teacher–child closeness was nonsignificant. Our findings are consistent with those of Portilla et al. (2014) in finding no bidirectional link between self-regulation and teacher–child conflict. However, our study extends this work by demonstrating that such reciprocal dynamics may instead operate across other interrelated domains of child functioning (Yang et al., 2021). They found that higher initial levels of teacher–child conflict were longitudinally associated with a decline in school engagement by the end of the kindergarten year. This association can be partly explained by the fact that children exhibiting inattentive and impulsive behaviors often struggle to form positive relationships with teachers (Campbell, 2000). Second, teachers may perceive children who lack self-regulatory skills as intentionally misbehaving, which can elicit disciplinary responses and exacerbate conflict. Furthermore, interactions may become limited to instructional exchanges, thereby reducing opportunities for positive mutual engagement (Yang et al., 2021; Rudasill et al., 2011).

Our findings also indicate that initiative negatively correlates with teacher–child conflict, whereas the degree of closeness relationship with children yielded insignificant results. The findings can be understood within the interpersonal cognition theory, where initiative represents a construct of proactive self-regulation; children develop the basis for complex metacognitive processes, such as seeking, understanding, and maintaining relationships (Warner et al., 2017). This is also a process of interpersonal cognition, the set of mental processes by which children think about their interactions and relationships with others (Bin, 2024). In interpersonal situations, children with higher psychological resilience are more likely to have the interpersonal cognitive advantage to invoke social support resources. In such situations, children with greater psychological resilience are more inclined to possess the cognitive edge of mobilizing social support resources. If children accurately assess interpersonal dynamics, they can engage in appropriate behavior to establish the relationship when asking for the help of others, and they will actively seek help through trusting interpersonal relationships.

### 5.2 Relationships between teacher–child relationships and behavioral problems

Our study found that teacher–child closeness in Wave 1 was negatively associated with behavioral problems at the same time point. In contrast, teacher–child conflict at both waves was positively associated with behavioral problems at Wave 2. These results suggest that teacher–child closeness may function as a protective factor, while conflict may serve as a risk factor for behavioral problems. Although the correlational design precludes definitive causal conclusions, these findings align with prior longitudinal research (Paes et al., 2023; Berry and O'Connor, 2010; Skalická et al., 2015). For children in small groups (i.e., ≤ 15 children), greater closeness was longitudinally linked to fewer behavioral problems in first grade (Blair and Raver, 2012). For left-behind children in particular, these findings carry notable practical implications. Given their limited access to parental support, positive teacher–child relationships may serve as a particularly important protective buffer against behavioral difficulties. Consequently, interventions aimed at enhancing teacher–child relationship quality in preschools serving left-behind children may yield disproportionately large effects in reducing behavioral problems within this high-risk population.

### 5.3 Mediation of self-regulation

Our study found that self-regulation in wave 1 fully mediated the relationship between teacher–child closeness in wave 1 and behavioral problems in wave 2. In contrast, self-regulation in wave 1 partially mediated the effect of teacher–child conflict in wave 1 on behavioral problems in wave 2. Furthermore, self-regulation in wave 2 partially mediated the effect of teacher–child conflict in wave 2 on behavioral problems in wave 2. These findings are consistent with theoretical perspectives and related empirical evidence. From a theoretical perspective, self-regulation is a complex, multi-component construct defined as the monitoring and controlling of all aspects of human behavior, including emotional, social, and motivational elements (Blair and Raver, 2012; Montroy et al., 2016). In contrast to self-regulation, which pertains to individual behavioral control, both coregulation and socially shared regulation are collaborative processes that require the mutual adjustment of regulatory efforts between individuals (Zachariou and Whitebread, 2019). Consistently, evidence documented that teacher–child conflict was negatively related, and teacher–child closeness was positively related to self-regulation among children in kindergarten. Children's self-regulation can predict their behavioral problems (Lonigan et al., 2017). There is evidence that young children's social performance, such as behavioral control and emotion regulation, is related to various aspects of self-regulation (Dias and Cadime, 2017; Ceylan and Asi, 2022). Our findings expand the literature by demonstrating that the mediating role of self-regulation is relevant to the teacher–child relationship and behavioral problems and that the mediation process holds for preschool children. Given that many left-behind children are raised by grandparents with limited knowledge of child development, teacher scaffolding of self-regulation via structured activities and predictable routines is particularly effective in reducing behavioral problems.

Notably, among the three resilience dimensions, only self-regulation emerged as a significant mediator, whereas initiative and attachment/relationships did not. Viewed through an attachment-theoretical lens, this pattern can be elucidated by the developmental specificity inherent to each dimension. Self-regulation—the capacity to modulate emotions and behaviors—is directly cultivated through daily teacher–child interactions, wherein teachers provide scaffolding, behavioral guidance, and emotional co-regulation (Blair and Raver, 2012). In contrast, initiative reflects a more dispositional tendency toward independent action, which may be less susceptible to relational influences within a 1-year timeframe. Similarly, the attachment/relationships dimension encompasses a broad relational capacity spanning multiple contexts (e.g., peers, family, and teachers); consequently, it is less contingent upon the dynamics of a single teacher–child dyad. Thus, self-regulation likely represents the most proximal mechanism linking teacher–child relationship quality to behavioral outcomes in this age group.

## 6 Conclusions

Several limitations within our study warrant attention. First, the cross-lagged panel design offers advantages over cross-sectional studies in discerning the directional relationships between variables; however, these associations do not imply causality between child resilience and teacher–child relationships. Further research is required to explore these associations. Second, the results are subject to method biases, as only teachers reported about children's resilience. Social desirability biases may lead teachers to inflate the measurement error when completing questionnaires because family factors may also influence children's resilience (Campbell et al., 2021). Future studies should collect parent- and teacher-reported data to evaluate children's resilience. Third, several sample limitations and characteristics diminish the generalizability of the findings. Our sample population primarily consisted of individuals from families with low socioeconomic status, necessitating further validation to determine the generalizability of the results. Future studies should approach broader demographics, including urban children and those from ethnic minorities. An increasing body of research highlights the significance of teacher–child relationships in shaping children's development (Verschueren, 2015). Our findings highlight how resilience affects children's teacher–child relationships, which have been largely overlooked. Promoting and intervening in children's resilience could have a broader, positive effect on their relationships with teachers. Furthermore, our findings provide critical insights into the factors that contribute to the positive development of behavioral problems and the influence of psychological resilience on teacher–child relationships and children's behavioral problems. Differences in the development of behavioral problems were already present in early childhood (Chen, 2024), and were longitudinally associated with teacher–child relationships and self-regulation and affected children's positive development. Collectively, these findings could be interpreted as providing support for preventing behavioral issues, underscoring the proactive role that preschool children take in their development. Specifically for left-behind preschool children, these findings highlight three critical imperatives: training teachers to foster warm, supportive relationships that actively cultivate self-regulation; implementing classroom-based interventions targeting self-regulatory skills; and acknowledging the bidirectional nature of these dynamics, wherein children's inherent resilience capacities shape their relational experiences with teachers. Collectively, these insights advocate for comprehensive prevention strategies that simultaneously address relational quality and individual resilience within early childhood education for vulnerable populations.

## Data Availability

The original contributions presented in the study are included in the article/supplementary material, further inquiries can be directed to the corresponding author.
