# Correction: Longitudinal associations between teacher–child relationships, resilience, and behavioral problems among preschool children

**DOI:** 10.3389/fpsyg.2026.2002583

**Published:** 2026-09-28

**Authors:** Lanlan Luo, Xiaoxia Fu, Shan Jin, Yanzhen Xu

**Affiliations:** 1 School of Educational Science, Guangdong Polytechnic Normal University, Guangzhou, China; 2 Business School, Central University of Finance and Economics, Beijing, China; 3 Institute for the Rule of Law and Standards in Education, China National Academy of Educational Sciences, Beijing, China

**Keywords:** behavioral problems, cross-lagged models, preschool children, resilience, teacher–child relationships

In the published article, the name of the fourth author was published incorrectly as “Yonzhen Xu”. The correct spelling is “Yanzhen Xu”.

In the published article, the affiliation for author Yanzhen Xu was published incorrectly as “Guangdong Technology Normal University, Guangzhou, China”. The correct affiliation is “School of Educational Science, Guangdong Polytechnic Normal University, Guangzhou, China”.

In the published article, a funding source was omitted from the **Funding** statement. The following has been added: “This work was supported by the Guangdong Philosophy and Social Science Planning Project 2025 (Grant No. GD25CJY13).”

The original version of this article has been updated.

